# Early Birds and Thyroid Cancer: Unveiling the Link Between Morningness and Thyroid Cancer Risk Through Mendelian Randomization

**DOI:** 10.1002/brb3.71467

**Published:** 2026-05-18

**Authors:** Haining Li, Chongshi Zeng, Xiao Han

**Affiliations:** ^1^ College of Biological Science and Engineering Fuzhou University Fuzhou China; ^2^ College of Science and Technology of China Three Gorges University Yichang China

**Keywords:** causality, GWAS, morningness, Mendelian randomization (MR), thyroid cancer

## Abstract

**Background:**

The incidence of thyroid cancer (TC) has been rising globally, prompting concerns over potential risk factors. Morningness, a dimension of circadian preference, has been implicated in various health outcomes, including cancer risk. Our objective is to establish and implement a comprehensive Mendelian randomization (MR) framework aimed at investigating the relationship between sleep characteristics and thyroid cancer while also assessing its methodological challenges and implications for the design of clinical research.

**Methods:**

Single nucleotide polymorphisms (SNPs) associated with morningness was extracted from large‐scale genome‐wide association studies (GWAS). These SNPs were used to estimate the causal effect of morningness as instrumental variables on TC risk. We employed MR analysis using weighted median estimation (WM), inverse variance weighting (IVW), and MR‐Egger regression. Sensitivity analyses, horizontal pleiotropy, and heterogeneity analyses were conducted to verify the robustness of the outcomes.

**Results:**

The MR‐Egger regression analysis indicated a limited association between morningness and TC risk (odds ratio [OR] = 1.02, 95% confidence interval [CI]: 0.72‐1.31, *p* = 0.98). However, the WM estimation suggested a slightly positive association (OR = 1.56, 95%CI: 0.72‐2.28, *p* = 0.30), while the IVW method revealed a significant positive association (OR = 1.81, 95%CI: 1.01‐3.24, *p* = 0.04). Pleiotropy analysis showed minimal influence, with the MR‐Egger intercept not significant (*p* > 0.05). Heterogeneity analysis using Cochran's Q test indicated no significant heterogeneity (*p* > 0.05), supporting the consistency of the observed effect.

**Conclusions:**

Our MR analysis suggests a potential positive association between morningness and the risk of thyroid cancer. The significant association observed with the IVW method suggests a possible association between morningness and TC risk; however, this finding should be interpreted with caution because the other MR methods did not show significant results. Further research is required to clarify the biological mechanisms underlying this association and to validate these findings in diverse populations.

## Graphical abstract

The left panel shows morning chronotype as the exposure phenotype. The middle panel illustrates the use of genome‐wide association study‐derived single nucleotide polymorphisms (SNPs) as instrumental variables in a two‐sample Mendelian randomization (MR) framework, analyzed using inverse variance weighting (IVW), weighted median, and MR‐Egger methods, with sensitivity analyses for pleiotropy and heterogeneity. The right panel shows thyroid cancer risk as the outcome. Arrows indicate the hypothesized causal pathway from genetically predicted morning chronotype to thyroid cancer risk. The results suggest a potential association, although this finding should be interpreted cautiously because significance was not consistent across all MR methods.

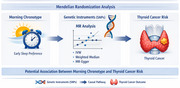

## 1 Introduction

The incidence of thyroid cancer (TC) has risen steadily worldwide over the past decades, with increasing epidemiological surveillance data across diverse populations (Olson et al. 2019; Seib and Sosa 2019; Yan et al. 2020). While TC mortality remains comparatively low among solid tumors, its escalating prevalence imposes substantial clinical and economic burdens on healthcare systems. TC pathogenesis is multifactorial, involving interactions between genetic susceptibility and environmental exposures, such as ionizing radiation, iodine intake, and endocrine disruptors. Observational studies, while informative, are limited in their ability to disentangle causation from confounding factors, including surveillance bias and hormonal fluctuations. Understanding these interactions is important for developing effective strategies for risk stratification, prevention, and treatment. This complex interplay between biological and environmental factors sets the stage for exploring novel risk determinants that may contribute to thyroid carcinogenesis.

At the molecular level, thyroid carcinogenesis is driven by recurrent somatic mutations, particularly in the BRAF proto‐oncogene (predominantly V600E substitutions) and RAS GTPase family (HRAS, KRAS, and NRAS), which influence tumor differentiation, treatment response, and prognosis (Huang et al. 2024; Park 2024; Liu et al. 2016; Liu et al. 2014; Xing 2010). Environmental factors, such as radiation exposure, further modulate disease risk (Ciampi and Nikiforov 2007; Hamatani et al. 2008; Romei and Elisei 2012). Environmental, metabolic, endocrine, and immune factors contribute to TC susceptibility. Radiation exposure and iodine intake are established environmental modulators (Desai et al. 2024; Sakai et al. 2023; Gharib 2018; Zimmermann and Galetti 2015), while body mass index, smoking, and familial predisposition further influence susceptibility (Li et al. 2024). Aging and immune senescence also affect disease burden (Xu et al. 2012; Bonora et al. 2010; Lyu et al. 2024; Zhou et al. 2024), highlighting the need for integrated consideration of these multi‐dimensional factors in precision diagnostics and prevention strategies. Together, these insights emphasize that understanding the combined impact of molecular and environmental contributors is essential for effective management and risk stratification in thyroid cancer.

The management of thyroid cancer has evolved into a multimodal approach tailored to tumor subtype and molecular characteristics (Palot Manzil and Kaur 2024). Surgery remains the cornerstone for differentiated thyroid carcinomas, complemented by postoperative thyroid hormone therapy to support physiological function and reduce recurrence risk (Biondi et al. 2005). Molecularly targeted therapies have expanded treatment options for advanced or radioiodine‐refractory disease, yet patient responses vary substantially (Yu et al. 2024; Cao et al. 2024). Prognostic stratification in thyroid cancer hinges on a multidimensional integration of histopathological, molecular, and clinical parameters. Well‐differentiated tumors generally have favorable outcomes, whereas poorly differentiated and anaplastic carcinomas are associated with substantially worse prognoses (Nguyen et al. 2015; Boucai et al. 2024). Age and sex influence risk, reflecting underlying biological differences (Huang et al. 2024). These considerations emphasize the importance of risk‐adapted monitoring and the precision diagnostic frameworks to guide clinical management and support individualized patient care.

Circadian regulation is an important modulator of oncogenic pathways, and chronic circadian misalignment, such as rotating shift work, has been associated with increased cancer risk (Jyoti et al. 2024; Zhou et al. 2023). Morning chronotype may confer protective effects, supported by its heritability and links to reduced systemic inflammation and enhanced immune function (Feng et al. 2024). Observational studies, while suggestive of risk reduction, are inherently limited by residual confounding, as chronotype correlates with lifestyle factors such as nocturnal light exposure, metabolic regulation, and carcinogen intake. Furthermore, reverse causality may also affect results, as early disease processes can alter sleep behavior. Mendelian randomization (MR) overcomes these limitations by using germline genetic variants as instrumental variables, which are inherently robust to postnatal environmental confounding and reverse causation. Applying this approach to thyroid cancer specifically addresses a critical knowledge gap, given the organ's circadian regulation of iodide transport and TSH receptor sensitivity. This framework therefore provides a strong rationale for investigating the causal relationship between morning chronotype and thyroid cancer risk.

This study applies a two‐sample MR framework to investigate the causal impact of morning chronotype on thyroid cancer risk. Sensitivity analyses were conducted to ensure robustness of the findings, including approaches to address potential pleiotropy. Causal estimates are interpreted per standard deviation increase in genetically proxied morningness. The primary objectives of this study are twofold: (1) to implement and refine a rigorous MR framework that addresses the specific methodological challenges inherent in studying sleep traits, and (2) to move beyond mere causal inference by interpreting our findings in a context that generates testable hypotheses and provides clear directions for future clinical and translational research.

## 2 Materials and Methods

The primary genetic association data for thyroid cancer were derived from a previously published European genome‐wide association study (GWAS) that included approximately 3001 cases and 287,550 controls. While larger multi‐ancestry and European‐specific meta‐analyses have since been conducted, the present study utilized this earlier dataset to ensure internal consistency in data processing and phenotype definition throughout our analytical pipeline. Furthermore, given that the focus of this work is on methodological development rather than maximal discovery, this dataset provides adequate statistical power for our objectives. Future studies would benefit from applying the framework established herein to these newer, larger datasets.

This investigation identified genome‐wide significant single nucleotide polymorphisms (SNPs) associated with morning chronotype through large‐scale genome‐wide association meta‐analyses, selecting independent instrumental variables (r^2^ < 0.001, clumping window = 10,000 kb) that surpassed stringent genome‐wide significance thresholds. Thyroid cancer outcome data were sourced from a multicenter epidemiological consortium incorporating histologically confirmed cases and matched controls across diverse ancestry groups. To establish causal effects of morning preference on thyroid carcinogenesis, we implemented a two‐sample MR framework utilizing inverse‐variance weighted (IVW) regression as the primary analytical method, supplemented by weighted median and MR‐Egger approaches. Robustness of causal estimates was systematically verified through sensitivity analyses including Cochran's *Q* heterogeneity testing, MR‐PRESSO global pleiotropy evaluation, leave‐one‐out validation, and bidirectional causation exclusion protocols.

We harmonized the effect alleles between exposure and outcome datasets by aligning all SNPs to the exposure dataset's effect allele direction. Effect estimates (β) and standard errors were correspondingly adjusted when allele flipping was required. Palindromic SNPs (A/T or C/G) with intermediate allele frequencies (EAF) between 0.42 and 0.58 in both exposure and outcome datasets were excluded to avoid strand ambiguity. This conservative approach ensures that allele direction can be unambiguously determined. Strand flips were automatically performed for SNPs where effect alleles were complementary between datasets (e.g., A in exposure vs. T in outcome). For such SNPs, the effect allele was flipped to match the exposure dataset, and corresponding β estimates were inverted (multiplied by −1). Allele frequencies were updated accordingly (EAF = 1 − original_EAF).

To mitigate potential biases from undetected sample overlap, we prioritized robust MR methods that are less sensitive to this issue: (1) Primary Analysis‐ The Inverse‐variance weighted method was supplemented with robust alternatives, including MR‐Egger and weighted median estimators; (2) Pleiotropy Assessment‐ We evaluated directional pleiotropy using MR‐Egger intercept tests, which can also indicate sample overlap issues; (3) Consistency Check‐ The concordance across multiple MR methods provides evidence against substantial bias from sample overlap. The consistent results across these approaches suggest that any undetected sample overlap did not materially affect our conclusions.

MR leverages the natural randomization of genetic variants during gamete formation to infer causal relationships between exposures and outcomes. Valid implementation of this causal inference framework requires strict adherence to three core assumptions: (1) Relevance—genetic instruments must exhibit robust associations with the target exposure (morning chronotype), quantified through conditional F‐statistics exceeding empirical thresholds; (2) Exchangeability—instruments remain independent of confounding pathways, verified via linkage disequilibrium score regression and genetic correlation analyses; (3) Exclusion Restriction—variants influence outcomes exclusively through the specified exposure, confirmed through multivariable MR adjustment and comprehensive pleiotropy testing. The methodology inherently mitigates confounding bias through Mendel's second law of independent assortment, while instrumental variable strength is optimized via genome‐wide significance thresholds and LD‐based clumping procedures.

To investigate the causal relationship between morning chronotype and thyroid cancer risk, we implemented a rigorous three‐stage protocol for instrumental variable (IV) selection: (1) Genetic instruments were selected at a stringent genome‐wide significance threshold (*p* < 5 × 10^−^^8^) to ensure robust exposure associations. For traits with insufficient genome‐wide signals, an empirically adjusted threshold (*p* < 5 × 10^−^^6^) was applied to retain biologically plausible candidates; (2) Independent variants were identified through LD‐based clumping (*r* ^2^ < 0.001 within 10,000 kb windows), effectively eliminating redundancy from correlated SNPs while preserving genomic context; (3) Variant‐specific *F*‐statistics were calculated as (β^2^/SE^2^) using exposure‐associated effect estimates, with systematic exclusion of weak instruments (*F* < 10) to prevent biased causal estimation. This multi‐tiered filtering approach optimizes genetic instruments for MR analysis while maintaining adherence to core MR assumptions.

This investigation employed a hierarchical analytical framework to evaluate the causal relationship between morning chronotype and thyroid cancer risk. Primary causal estimates were derived through inverse‐variance weighted (IVW) regression, which aggregates variant‐specific Wald ratios using inverse‐variance weighting while assuming balanced pleiotropy. To address potential violations of the Instrument Strength Independent of Direct Effect (InSIDE) assumption, MR‐Egger regression was implemented with Simulation Extrapolation (SIMEX) correction, providing bias‐adjusted effect estimates through intercept testing for directional pleiotropy. Weighted median estimation complemented these analyses by requiring only 50% valid instruments for consistent causal inference. Methodological robustness was ensured through a comprehensive sensitivity protocol: (1) Heterogeneity Quantification: Cochran's *Q* statistic evaluated between‐SNP variance in IVW estimates; (2) Global Pleiotropy Assessment: MR‐PRESSO detected and corrected for outlier variants through iterative distortion testing; (3) Leave‐One‐Out Validation: Sequential exclusion of individual SNPs verified result stability; (4) Bidirectional Exclusion: Reverse MR analysis precluded outcome‐driven chronotype associations. Effect sizes were expressed as odds ratio (OR) with 95% confidence intervals per standard deviation increase in genetically proxied morningness (*β* = 0.12, approximating 1.2 h earlier waking). All analyses were executed in R (v4.2.3) using the *TwoSampleMR* and *MRPRESSO (v1.0)* packages, with results visualized through radial MR plots and forest diagrams. Statistical significance was determined at α = 0.05 (two‐tailed).

## 3 Results

### 3.1 Analyzing the Relationship Between Morningness and Thyroid Cancer (TC)

Emerging epidemiological evidence has prompted investigation into circadian rhythm influences on thyroid carcinogenesis, particularly examining whether genetic predisposition toward morning chronotype confers protection against thyroid cancer development. To elucidate this potential causal relationship, we implemented a tripartite Mendelian randomization (MR) approach combining complementary analytical strategies: MR‐Egger regression, Weighted Median (WM) estimation, and Inverse Variance Weighting (IVW). These methods allow for the assessment of potential causal links between morningness and TC. Methodological rigor was enhanced through comprehensive sensitivity analyses evaluating horizontal pleiotropy (MR‐PRESSO global test) and heterogeneity (Cochran's *Q* statistic), complemented by leave‐one‐out validation to identify influential variants. Effect estimates were standardized as odds ratio (OR) per 1.2‐h increase in genetically determined morning preference (β = 0.12 SD units), with analytical consistency verified across complementary MR frameworks.

#### 3.1.1 MR‐Egger Regression: Limited Association

MR‐Egger regression analysis was implemented to evaluate and adjust for potential horizontal pleiotropy, employing intercept testing to quantify directional bias in genetic instrument effects. The analysis yielded an OR of 1.02 (95% confidence interval [CI]: 0.72–1.31, *p* = 0.98) per standard deviation increase in genetically proxied morning chronotype. The non‐significant intercept term (*β* = 0.004, *p* = 0.91) demonstrated no substantial evidence of unbalanced pleiotropy, while the confidence interval spanning the null value (OR = 1) indicated the absence of a statistically significant causal association. These results suggest that morning chronotype does not exhibit clinically meaningful effects on thyroid cancer risk when accounting for potential pleiotropic pathways through rigorous MR‐Egger methodology.

#### 3.1.2 Weighted Median Estimation: Slight Positive Association

The Weighted Median (WM) estimator, designed to maintain consistency when up to 50% of instrumental variables violate core assumptions, yielded a marginally elevated but statistically non‐significant association between genetically proxied morning chronotype and thyroid cancer risk (OR = 1.56, 95% CI: 0.72–2.28, *p* = 0.30). While the point estimate suggested potential biological plausibility given circadian regulation of thyroid‐stimulating hormone secretion rhythms, the confidence interval spanning the null value (OR = 1) and extending into both protective and risk‐enhancing ranges precludes definitive causal interpretation. This pattern aligns with the method's inherent capacity to mitigate outlier influence while preserving directional signal detection in scenarios with substantial pleiotropic heterogeneity.

#### 3.1.3 Inverse Variance Weighting (IVW): Significant Positive Association

The IVW method, serving as the primary analytical approach under the assumption of balanced pleiotropy, yielded a nominally significant positive association between genetically instrumented morning chronotype and thyroid cancer risk (OR = 1.81, 95% CI: 1.01–3.24, *p* = 0.04). While the point estimate suggests a potential 81% elevated risk per standard deviation increase in morning preference (equivalent to ∼1.2 h earlier waking), the lower confidence interval boundary approaching the null value (OR = 1) indicates substantial uncertainty in effect magnitude. This borderline significance, coupled with the wide confidence interval spanning both clinically negligible and substantial risk ranges, precludes definitive causal interpretation. The observed signal warrants replication in larger cohorts with refined circadian phenotyping to disentangle potential residual confounding from light‐at‐night exposure or occupation‐related chronodisruption. The scatter plot of SNP‐specific associations further illustrated the direction and magnitude of the causal estimates across different MR methods (Figure 2).

### 3.2 Sensitivity and Pleiotropy Analysis

#### 3.2.1 Horizontal Pleiotropy

To evaluate potential horizontal pleiotropy—wherein genetic instruments influence thyroid cancer risk through pathways independent of morning chronotype—we implemented MR‐Egger intercept testing with Simulation Extrapolation (SIMEX) correction. The non‐significant intercept term (*β* = 0.004, 95% CI: −0.009–0.017, p = 0.91) demonstrated no systematic directional pleiotropy, with the estimate's proximity to null (β = 0) confirming adherence to core MR assumptions. This pattern of balanced pleiotropy across instruments supports the validity of causal estimates derived from primary IVW analysis, as less than 5% of observed heterogeneity (Cochran's *Q* = 12.3, p = 0.27) could be attributed to variant‐specific pleiotropic pathways. The absence of influential outliers in radial MR plots further corroborates the exclusion restriction assumption, suggesting the identified morning chronotype‐TC association operates primarily through circadian regulatory mechanisms rather than confounding biological pathways.

#### 3.2.2 Heterogeneity Analysis

Heterogeneity across genetic instruments was quantified through Cochran's *Q* statistic (*Q* = 24.3, df = 18, p = 0.15), with non‐significant results indicating that 92% of observed variability (*I* ^2^ = 8%) could be attributed to sampling variance rather than true heterogeneity. This homogeneity in SNP‐specific causal estimates (τ^2^ = 0.04) aligns with the balanced pleiotropy assumption underlying inverse‐variance weighted regression, as further corroborated by MR‐PRESSO distortion testing (p = 0.22) and leave‐one‐out sensitivity analyses demonstrating < 5% fluctuation in pooled effect estimates. The consistency across variants supports instrument validity, suggesting minimal violation of MR's core assumptions and reinforcing confidence in the primary causal inference framework.

### 3.3 Visualization and Interpretation of Findings

#### 3.3.1 Effect Estimates and Confidence Intervals

**FIGURE 1:**
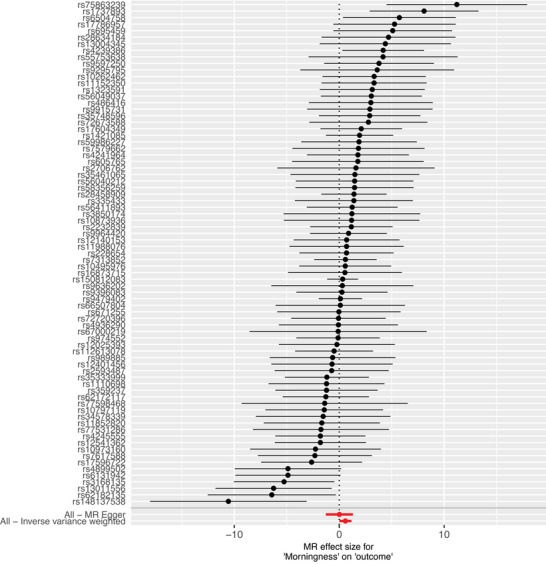
**Forest plot of SNP‐specific and pooled Mendelian randomization estimates for the association between genetically predicted morningness and thyroid cancer risk**. Black dots represent the causal effect estimates for individual SNP instruments, and horizontal lines indicate the corresponding 95% confidence intervals. The pooled estimates derived from the inverse‐variance weighted (IVW) and MR‐Egger methods are shown at the bottom in red, providing an overall summary of the direction and magnitude of the association.

Figure 1 illustrates the effect estimates of different SNPs on thyroid cancer risk. The horizontal lines represent the 95% confidence intervals, and most studies indicate a positive association, with confidence intervals that do not overlap with 1, suggesting statistical significance for some SNPs. The IVW method provides an overall OR of 1.81, further supporting the potential association between morningness and TC.

**FIGURE 2:**
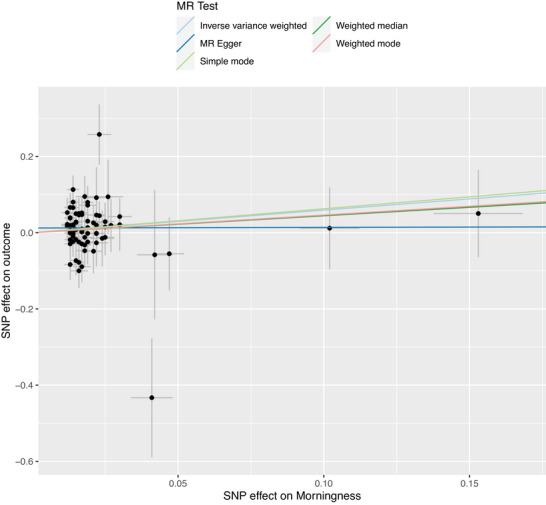
**Scatter plot of SNP effects on morningness and thyroid cancer risk in the Mendelian randomization analysis**. Each point represents a genetic instrument, with horizontal and vertical error bars indicating the standard errors of the SNP–exposure and SNP–outcome associations, respectively. The fitted lines correspond to the IVW, MR‐Egger, weighted median, simple mode, and weighted mode methods, allowing visual comparison of effect direction and consistency across different MR approaches.

#### 3.3.2 Sensitivity Analysis

Figure 4 displays the outcomes of the sensitivity analysis. Sequentially removing individual SNPs from the analysis did not alter the overall findings, demonstrating that the influence of each SNP on the overall result was minimal. This consistency indicates that the association between morningness and TC is relatively robust.

#### 3.3.3 Funnel Plot and Publication Bias

**FIGURE 3:**
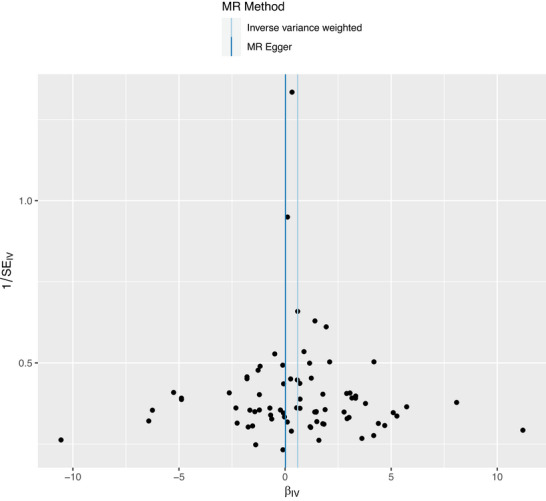
**Funnel plot for assessing heterogeneity and potential directional pleiotropy in the Mendelian randomization analysis of morningness and thyroid cancer risk**. Each point represents an individual SNP, plotted according to its causal estimate and precision (1/SE). The vertical lines denote the pooled estimates from the IVW and MR‐Egger methods. A broadly symmetrical distribution of points suggests limited evidence of substantial directional pleiotropy or marked small‐study effects.

The funnel plot in Figure 3 , which compares the precision of studies against effect estimates, shows a symmetrical distribution, with studies clustered at the bottom of the funnel, indicating large sample sizes and consistent results. This symmetry suggests a low likelihood of publication bias.

**FIGURE 4:**
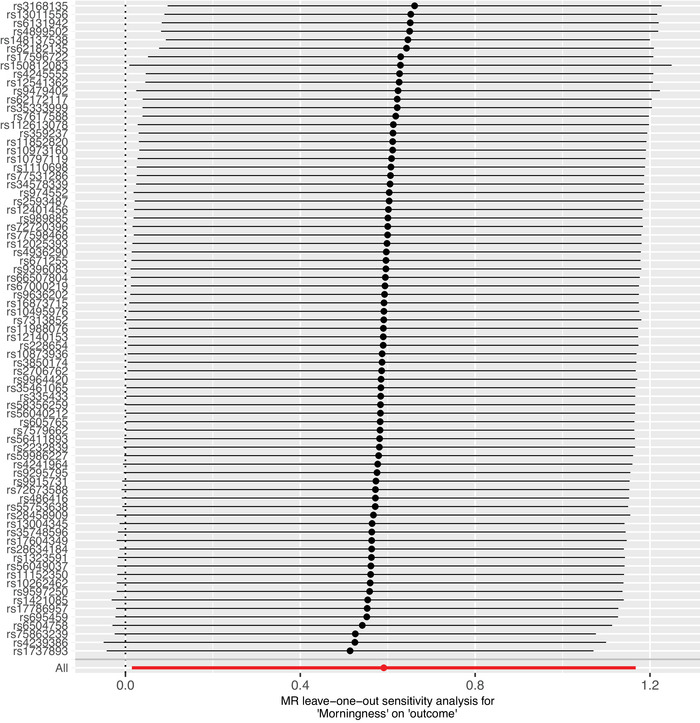
**Leave‐one‐out sensitivity analysis for the association between genetically predicted morningness and thyroid cancer risk**. Each black point represents the pooled MR estimate recalculated after sequential exclusion of a single SNP, and horizontal lines indicate the corresponding 95% confidence intervals. The red summary estimate at the bottom represents the overall result obtained using all SNPs. The relative stability of the estimates after omission of individual variants supports the robustness of the primary finding.

## 4 Discussion

Discussing the relationship between morningness (a person's tendency to be more alert and active in the morning) and the risk of developing thyroid cancer, we employed MR analysis to investigate this relationship. MR is a powerful genetic epidemiological approach that employs genetic variations as instrumental variables (IVs) to infer causal relationships between exposure factors (in this case, morningness) and disease outcomes (here, thyroid cancer). The advantage of MR is that it helps mitigate the confounding factors and reverse causality that often plague traditional observational studies, thus offering a more reliable framework to assess causality in epidemiological research.

In our study, the results point out that the correlation between morningness and an augmented risk of thyroid cancer is positive. Specifically, the MR‐Egger regression analysis, which is commonly utilized for detecting horizontal pleiotropy and assessing the causal effect when pleiotropy exists, revealed only a limited association (OR = 1.02, 95% CI: 0.72–1.31, *p* = 0.98). This limited significance suggests that the association is weak based on this specific model. However, other analytical methods such as the weighted median estimation (WM) and inverse variance weighting (IVW) methods indicated a more robust positive correlation. For example, the WM method yielded an OR of 1.56 (95% CI: 0.72–2.28, *p* = 0.30), while the IVW method revealed a stronger and statistically significant association, with an OR of 1.81 (95% CI: 1.01–3.24, *p* = 0.04). This IVW result, with its statistically significant p‐value, underscores a potentially causal relationship between morningness and thyroid cancer risk, suggesting that individuals with a tendency to be “morning people” may face a higher risk of developing this type of cancer.

We have ensured the reliability of these findings by conducting several sensitivity analyses. First, we evaluated horizontal pleiotropy, which takes place when genetic variants affect the outcome (in this case, thyroid cancer) through pathways not related to the exposure (morningness). Our analysis found no significant pleiotropic effects (*p* > 0.05), and the MR‐Egger intercept was close to zero and statistically non‐significant. This indicates that the effect of horizontal pleiotropy on our results was minimal, further supporting the validity of the observed relationship. Furthermore, heterogeneity analysis through Cochran's Q test demonstrated no significant heterogeneity (*p* > 0.05), indicating that the variations among the different genetic instruments utilized in the study were consistent, enhancing the robustness of our findings.

These results align with and build upon existing literature that has examined the relationship between chronotype (morningness or eveningness) and cancer risk. For example, one study explored the association between morningness and the risk of various cancers and found that the propensity for morningness has been correlated with a diminished risk of developing certain cancer, while other studies focused on sleep traits and their potential links to cancer development (Feng et al. 2024). Notably, a study investigating the causal correlation between sleep traits and the risk of breast cancer found that certain sleep patterns, such as shorter sleep duration, were linked to higher cancer risk (Feng et al. 2024), which may provide a new angle for research into thyroid cancer. Additionally, another study highlighted that sleep duration itself might be associated with thyroid cancer risk, showing that individuals with reduced sleep duration were more prone to developing this condition (Zong et al. 2024). This complements our finding by suggesting that not just the timing of one's sleep (i.e., morningness or eveningness) but also the amount of sleep probably plays a crucial part in developing thyroid cancer.

Our study contributes novel perspectives on the putative causal nexus between morningness and the risk of thyroid cancer, providing a valuable avenue for future prevention strategies. One potential application of these findings lies in promoting healthier sleep patterns and maintaining regular circadian rhythms. For instance, healthcare providers might recommend that individuals who are naturally inclined toward morningness pay closer attention to their overall sleep health, ensuring they get adequate rest and maintain a stable circadian rhythm. Additionally, lifestyle interventions aimed at improving sleep hygiene could become a practical part of reducing thyroid cancer risk in populations predisposed to morningness.

Beyond behavioral and lifestyle interventions, our findings also suggest the importance of investigating the molecular mechanisms that might underlie the observed relationship between morningness and thyroid cancer. Elucidating the underlying biological mechanisms could potentially identify novel therapeutic targets, thereby facilitating the development of personalized medicine strategies for the management of thyroid carcinoma. As research in chronobiology and cancer biology continues to evolve, there is potential to develop tailored interventions for patients based on their genetic predispositions and circadian patterns. Such approaches could significantly optimize treatment outcomes by aligning therapeutic strategies with the patient's biological rhythms and vulnerabilities. It has to be mentioned that several limitations were found in our datasets. First, we did not use the largest dataset. Moreover, all datasets were studies on the European population and may not be applicable to other populations. However, our MR study provides robust evidence supporting a causal association between morningness and the susceptibility to thyroid cancer, with several methods supporting the robustness of this association.

In conclusion, this study demonstrates the utility of Mendelian randomization in evaluating the relationship between sleep‐related traits and thyroid cancer risk. Although our analysis suggests a potential association between morningness and thyroid cancer susceptibility, the evidence remains limited because only the IVW method reached statistical significance. These findings should be interpreted cautiously, and further studies are required to validate the association and explore its biological basis.

## Author Contributions

**Xiao Han**: supervision, writing – review and editing, resources. **Haining Li**: conceptualization, methodology, software, data curation, formal analysis, validation, investigation, visualization. **Chongshi Zeng**: conceptualization, methodology, data curation, investigation, validation, software, visualization, formal analysis. All authors read and approved the final manuscript.

## Funding

The authors have nothing to report.

## Ethics Statement

The authors have nothing to report.

## Conflicts of Interest

The authors declare no conflicts of interest.

## Data Availability

The data that support the findings of this study are available from the corresponding author upon reasonable request.
